# The relationship between distributed leadership and teachers' teaching enthusiasm: the chain mediation of teacher autonomy and organizational commitment

**DOI:** 10.3389/fpsyg.2026.1896309

**Published:** 2026-08-19

**Authors:** Yang Zhang, Yipu Hao

**Affiliations:** 1 College of Special Education, Leshan Normal University, Leshan, China; 2 School of Education, Central China Normal University, Wuhan, China

**Keywords:** conservation of resources theory, distributed leadership, organizational commitment, teacher autonomy, teaching enthusiasm

## Abstract

**Introduction:**

Teachers' teaching enthusiasm is a key factor positively associated with sustained engagement in professional practice, higher teaching quality, and student development. Unlike general job related affective states such as job satisfaction or work engagement, teaching enthusiasm specifically captures the intrinsic enjoyment, value endorsement, and sustained passion for the core act of teaching. The specific mechanisms by which distributed leadership is associated with teachers' teaching enthusiasm remain an issue requiring further exploration.

**Methods:**

Data were drawn from 1,319 junior high school teachers in Shanghai who participated in the OECD's 2024 Teaching and Learning International Survey. Validated scales assessed distributed leadership, teacher autonomy, organizational commitment, and teaching enthusiasm. Structural equation modeling and bootstrap analyses were employed to test direct and chain mediation effects.

**Results:**

Distributed leadership showed significant positive correlations with teaching enthusiasm, teacher autonomy, and organizational commitment. Both teacher autonomy and organizational commitment were positively associated with teaching enthusiasm, and teacher autonomy positively predicted organizational commitment. Teacher autonomy and organizational commitment not only independently mediated the relationship between distributed leadership and teaching enthusiasm but also exerted a significant chain mediating effect.

**Conclusion:**

These findings extend Conservation of Resources theory by demonstrating that distributed leadership can ignite teaching enthusiasm through two pathways: first by granting teachers greater autonomy, which in turn fosters organizational commitment and thereby sustains passion for teaching. Practically, school leaders and policymakers should promote empowering leadership, protect instructional autonomy, and cultivate a culture of shared commitment to build a virtuous cycle of resource accumulation that strengthens the teaching workforce.

## 1 Introduction

Teachers' teaching enthusiasm is significantly associated with the quality of the educational system's operation (Keller et al., 2016). Teaching enthusiasm encompasses teachers' recognition of the intrinsic value of their work, emotional investment, and a sustained sense of fulfillment in professional practice (Kunter, 2013). Existing research has confirmed the educational functions of teaching enthusiasm across multiple dimensions: at the teacher level, teaching enthusiasm drives teachers to actively explore innovative teaching methods, engage in continuous professional learning and reflective practice, and effectively mitigate the effects of occupational burnout (Frenzel et al., 2018; Kunter et al., 2011); At the student level, teachers' teaching enthusiasm is transmitted to classroom interactions through emotional contagion mechanisms, positively promoting students' learning motivation, academic engagement, and achievement (Frenzel et al., 2019; Keller et al., 2014). Therefore, exploring the mechanisms underlying and factors associated with teachers' teaching enthusiasm holds both theoretical value and practical significance for enhancing the quality of school education and the development of the teaching workforce.

Although distributed leadership has been associated with a range of teacher affective outcomes, including job satisfaction and wellbeing, teaching enthusiasm represents a theoretically distinct construct. It is domain-specific, directly tied to the act of teaching, and considered a core affective–motivational component of teacher professional competence (Kunter, 2013), with unique emotional contagion effects on students. Unlike a global evaluation of working conditions or a general state of work-related wellbeing, teaching enthusiasm pertains exclusively to the passion and intrinsic enjoyment derived from teaching activities themselves, making it a more proximal and instructionally consequential outcome. This distinctiveness warrants dedicated examination within leadership research.

Research on the factors associated with teaching enthusiasm has primarily focused on two levels: the individual and the environmental. At the individual level, this study focuses on teachers' psychological capital and professional quality involved in variables of self-efficacy, professional identity, and career motivation, which are examined (Taxer and Frenzel, 2022); at the environmental level, factors such as organizational climate, social support, and leadership style are considered (Collie et al., 2016; Zhang and Ye, 2024). Among environmental factors, school leadership significantly relates to teachers' psychological wellbeing as a core component (Doguş, 2026). Existing research has largely focused on transformational leadership or instructional leadership, or on the direct relationship of leaders' personal traits on teachers' teaching (Kilinç et al., 2024). However, as school organizational structures evolve toward flatter hierarchies, distributed leadership, as a leadership model emphasizing power sharing and collaboration, has garnered attention (Harris, 2008). Distributed leadership posits that leadership practice is a dynamic process involving the interaction among leaders, followers, and the context, rather than being centered on the individual (Spillane, 2006). Existing research indicates that distributed leadership can enhance teachers' professional learning, job satisfaction, and innovative behavior (Buyukgoze et al., 2024; Liu et al., 2021). However, whether distributed leadership is positively associated with teaching enthusiasm remains to be empirically tested.

Conservation of Resources (COR) Theory provides a robust analytical framework for explaining how distributed leadership is associated with teachers' teaching enthusiasm (Hobfoll, 1989). The core assumptions of this theory are that individuals tend to acquire, maintain, and protect valued resources; resources are interrelated and gain exhibit a spiral effect; the organizational environment constitutes a “resource channel” through which individuals acquire resources. From the perspective of COR theory, teachers' teaching enthusiasm does not arise spontaneously or result solely from individual traits, but it is a positive state that requires the continuous investment of psychological energy and emotional resources to sustain (Hobfoll, 2011). The empowerment practices, collaborative culture, and trusting relationships inherent in distributed leadership may be associated with teachers' teaching enthusiasm through various pathways for resource transmission (Gronn, 2002). However, the questions exactly which specific resource variables distributed leadership utilizes and through what sequence of transmission it is linked to teaching enthusiasm, still require systematic theoretical construction and empirical testing.

Specifically, this study focuses on two potential mediating variables: Teacher Autonomy and Organizational Commitment. Teacher Autonomy is defined as the decision-making authority teachers possess regarding teaching content and processes (Runhaar et al., 2013). From the perspective of COR theory, Teacher Autonomy is classified as a condition resource, organizational commitment is defined as teachers' emotional identification with and sense of belonging to the school (Meyer and Allen, 1991), and falls under personal-characteristic resources. Previous studies have examined the independent associations of distributed leadership on teacher autonomy (Lin, 2022) and organizational commitment (Yao and Ma, 2024; Liu and Werblow, 2019). Additionally, some studies have shown that teacher autonomy is positively associated with organizational commitment (Özdemir et al., 2024). However, no study has yet integrated these variables into a unified analytical framework to systematically examine the dual mediating roles of Teacher Autonomy and organizational commitment in the process by which distributed leadership is associated with teaching enthusiasm, as well as their chain-mediated relationship.

To address this research gap, this study grounded in COR theory and utilizing TALIS 2024 data from Shanghai teachers, constructs and tests a chained mediation model aimed at sequentially answering the following questions: RQ1: Is there a significant positive correlation between distributed leadership and teachers' teaching enthusiasm in China? RQ2: Do teacher autonomy and organizational commitment mediate the relationship between distributed leadership and teacher teaching enthusiasm in China?

This study offers the following incremental contributions to the existing literature. First, we introduce teaching enthusiasm as a novel outcome variable in distributed leadership research, thereby expanding its nomological network from predominantly cognitive and behavioral consequences (e.g., self-efficacy, innovative behavior, and job satisfaction) to the core emotional–motivational domain of teaching. We further clarify that teaching enthusiasm is conceptually distinct from general job attitudes and work engagement because it embodies the intrinsic affective and motivational energy directed specifically at teaching practice, which is a primary factor associated with student learning. Second, moving beyond the isolated examination of single mediators, we reveal the resource flow mechanism stipulated by COR theory, how condition resources (autonomy) are transformed into personal-characteristic resources (commitment) in a gain spiral, which ultimately is associated with teaching enthusiasm. This chain-mediated perspective deepens current understanding of the “black box” between distributed leadership and teacher affective outcomes. Third, utilizing the large-scale, representative TALIS 2024 data from Shanghai, we provide evidence from a Chinese educational context where hierarchical traditions coexist with collaborative practices, thus testing the cross-cultural applicability of distributed leadership theory and COR theory. Moreover, by grounding the analysis in China's current reform priorities, such as the implementation of new curriculum standards, the “double reduction” policy, and the national strategy to build a high-quality teaching workforce, this study aims to generate context-specific recommendations for school leadership development and teacher motivation policies.

## 2 Literature review and research hypotheses

### 2.1 The Chinese educational context

China's education system has long been characterized by a combination of centralized administration and localized implementation. Since the 1985 reform that introduced the “Principal Responsibility System,” school principals have been granted greater decision-making authority, yet schools still operate within a hierarchical framework overseen by education authorities (Hawkins, 2000; Walker et al., 2012). In recent decades, further decentralization and curriculum reforms have encouraged power-sharing, teacher participation, and collaborative governance at the school level. These trends have created fertile ground for distributed leadership to emerge, though its form often blends top-down allocation with bottom-up collegiality (Lu, 2024). Within this structure, how principals empower teachers, foster collaborative cultures, and share leadership has become a pivotal issue in understanding teachers' motivation and wellbeing (Yao and Ma, 2024).

Shanghai, as one of China's most economically and educationally advanced municipalities, provides a distinctive setting for studying distributed leadership. Shanghai has consistently topped international assessments such as PISA, and its teachers have demonstrated high levels of professional collaboration and engagement (Gong, 2025). The city's educational authorities have invested heavily in teacher professional development, school-based management, and leadership capacity-building (Liu and Hallinger, 2018). Given Shanghai's progressive reforms and its relatively supportive policy environment for school autonomy, these context-specific characteristics underscore the need to examine, using a sample of teachers from China, particularly Shanghai, how distributed leadership is associated with teaching enthusiasm through differentiated pathways such as teacher autonomy and organizational commitment. These context-specific characteristics underscore not only the need to examine how distributed leadership influences teaching enthusiasm through autonomy and commitment, but also the potential of such findings to inform China's ongoing efforts to reform school governance and enhance teacher professional vitality. For policymakers, understanding these mechanisms is essential to designing leadership training programs and school evaluation systems that foster a motivated and sustainable teaching force.

### 2.2 Conservation of resources theory

COR theory was first systematically proposed by Hobfoll (1989) and was initially intended to provide an integrative explanatory framework for the mechanisms of stress generation in organizational contexts. Hobfoll (2011) broadly defined resources as anything that an individual perceives as helpful in achieving their goals. This broad definition gives it cross-situational theoretical applicability (Hobfoll, 2011). Halbesleben et al. (2014) classified resources into four distinct types. First, material resources refer to those with physical form or monetary value, such as teaching equipment and wages. Second, condition resources create the prerequisites and opportunities for individuals to acquire other resources, including job autonomy and social support networks. Third, energy resources are intangible capital that helps individuals gain additional resources, including time, knowledge, skills, and social capital. Fourth, personal characteristic resources refer to an individual's relatively stable internal psychological traits, such as self-efficacy, self-esteem, optimism, and organizational commitment (Hobfoll, 2011; Hobfoll et al., 2018).

The core assumptions of COR theory can be summarized in three points. First, the resource preservation priority assumption, which is an individual's motivation to protect existing resources takes precedence over the motivation to acquire new resources; the negative impact of resource loss on an individual is greater in intensity and duration than the positive impact of resource gain (Hobfoll, 2011; Hobfoll et al., 2018). Second, the Resource Investment Hypothesis. Individuals must continuously invest in existing resources to prevent resource loss, recover from loss, or acquire new resources; individuals with more initial resources are more likely to invest in resources, thereby entering a virtuous cycle of resource appreciation (Hobfoll, 2011; Hobfoll et al., 2018). Third, the resource caravans hypothesis posits that resources do not exist in isolation but appear in interconnected clusters; the organizational environment in which an individual operates constitutes a “resource caravan” that can either nurture the accumulation of individual resources or accelerate their depletion (Hobfoll, 2011; Hobfoll et al., 2018).

As research has progressed, the scope of COR theory's application has expanded from stress to the study of positive organizational behavior. In positive organizational behavior research, COR theory has been used to explain the mechanisms underlying the generation of positive states such as innovative behavior, work engagement, and career wellbeing (Qian et al., 2024; Wright and Hobfoll, 2004). In research on educational organizations, COR theory has also been used to analyze the theoretical foundations of self-efficacy, job satisfaction, and innovative practices (Liu et al., 2021; Buyukgoze et al., 2024; Sha and Chang, 2025; Wang and Bai, 2025). The aforementioned studies indicate that COR theory provides an effective analytical tool for understanding how the organizational environment is associated with individuals' positive psychological states by providing resources.

Applying COR theory to this study, teachers' teaching enthusiasm can be conceptualized as a positive emotional state that requires support from diverse resources. The generation and maintenance of teaching enthusiasm are not merely individual psychological processes but are profoundly influenced by the organizational environment. As an institutionalized organizational condition, school leadership styles constitute a key environmental channel through which teachers acquire or lose resources (Hobfoll et al., 2018). Indeed, empirical studies have demonstrated that distributed leadership provides teachers with condition resources such as autonomy through empowerment and collaborative culture (e.g., Liu et al., 2021; Özdemir et al., 2023) and fosters personal-characteristic resources like organizational commitment (e.g., Bellibaş et al., 2024; Yao and Ma, 2024). These resource gains can subsequently promote teachers' teaching enthusiasm, which is in line with the resource investment and caravan assumptions of COR theory. The following section based on the COR theoretical framework will derive the study's hypotheses one by one,

### 2.3 Distributed leadership and teachers' teaching enthusiasm

The theoretical origins of distributed leadership can be traced back to distributed cognition and activity theory. Gronn (2002) defines distributed leadership as a “joint action,” in which leadership functions are collaboratively performed by multiple organizational members based on their expertise and task requirements, rather than being undertaken independently by a single leader. Spillane (2004) further proposed a “leadership practice” perspective on distributed leadership, emphasizing that leadership is not an individual role or position, but rather a practical activity occurring at the interface of leaders, followers, and the situation. Harris's (2008) review study identified three core characteristics of distributed leadership: power sharing, wherein decision-making authority diffuses from a few leaders to organizational members; collaboration, wherein interdependent, complementary, and synergistic working relationships form among organizational members; and fluid boundaries, wherein leadership roles dynamically adjust based on task contexts and member expertise rather than being fixed to formal positions.

In the Chinese educational context, distributed leadership has been adapted and integrated into local culture. Lu (2024) points out that distributed leadership in Chinese schools exhibits distinct characteristics: on the one hand, a top-down “allocation” model coexists with the Western bottom-up “emergence” model; on the other hand, the traditional culture of collectivism endows Chinese distributed leadership with stronger collaborative presuppositions and relational embeddedness. Research by Yao and Ma (2024) further indicates that even within the hierarchical framework of China's educational management system, distributed leadership can positively influence teachers' psychological wellbeing through empowerment and participatory mechanisms.

Teaching enthusiasm is one of the central themes in research on teachers' emotions. In their multidimensional model of teacher professional competence, Kunter (2013) defined teaching enthusiasm as a composite psychological state comprising teachers' cognitive endorsement of the value of teaching, their emotional intrinsic enjoyment of teaching activities, and the motivational drive that sustains their ongoing commitment. Unlike transient emotional fluctuations, teaching enthusiasm exhibits stability over time and consistency across contexts, constituting a core component of teachers' professional identity. A longitudinal study by Frenzel et al. (2018) demonstrated that teachers' teaching enthusiasm significantly predicts subsequent innovative teaching behaviors and students' academic engagement, and that influence exhibits long-term stability. In the present study, consistent with the TALIS 2024 operationalization, we treat teaching enthusiasm as a global unidimensional construct that reflects the core affective–motivational essence of teaching, while acknowledging the multidimensional nature discussed in the literature (Keller et al., 2016; Kunter, 2013).

While previous leadership studies have focused on teacher job satisfaction, work engagement, or burnout, teaching enthusiasm is distinctive in that it reflects teachers' intrinsic cognitive and emotional connection to teaching content and process, rather than a global evaluation of the work environment. This domain-specific and instruction-oriented nature makes it a uniquely valuable outcome for examining how school leadership can energize the core educational mission. Based on COR theory, the impact of distributed leadership on teaching enthusiasm can be explained through resource channel mechanisms. First, distributed leadership provides teachers with institutionalized opportunities to participate in school decision-making and instructional planning through empowerment practices. This participation in decision-making not only expands the scope of teachers' roles but also conveys recognition and respect for their professional value (Halbesleben et al., 2014). From the perspective of resource gains, empowerment enables teachers to acquire more conditional resources that influence their work environment, and these incremental resources can stimulate their teaching enthusiasm. Second, the atmosphere of open communication and teamwork fostered by distributed leadership constitutes an energetic resource. Professional interactions and mutual support among teachers can effectively reduce role ambiguity and interpersonal isolation in the workplace, thereby mitigating the risk of emotional exhaustion (Doguş, 2026). When teachers perceive substantial support from principals and colleagues, they expend fewer psychological resources in coping with work stress, and the emotional energy dedicated to teaching is preserved and accumulated, allowing them to maintain a high level of teaching enthusiasm (Frenzel et al., 2018). Based on this, the following hypothesis is proposed:

**H1: Distributed leadership is significantly positively correlated with teachers' teaching enthusiasm**.

### 2.4 The mediating role of teacher autonomy

Teacher autonomy is defined as the sense of control and influence that teachers perceive they have over curriculum content, teaching strategies, and professional decisions during the teaching process (Pearson and Moomaw, 2005). As a condition resource, teacher autonomy reflects the degree of self-determination and discretion teachers have in their professional activities. Recent research indicates that teacher autonomy is a key factor in enhancing job satisfaction (Skaalvik and Skaalvik, 2014), stimulating innovative teaching behaviors (Gong, 2025), and alleviating professional burnout (Ha et al., 2025).

The relationship of distributed leadership on teacher autonomy can be explained in terms of resource investment. By fostering a culture of power-sharing and collaboration, distributed leadership conveys to teachers trust in their professional judgment and respect for their independent decision-making. This trust and respect constitute a structural work resource, enabling teachers to perceive greater control over their work and professional autonomy within the school (Lin, 2022). A study by Liu et al. (2021) using Chinese schools as a sample confirmed that distributed leadership significantly enhances teacher autonomy through shared decision-making mechanisms. Song and Hsieh (2026) study also indicates that distributed leadership substantially expands teachers' autonomy in selecting instructional content and methods by promoting their participation in decision-making.

As a condition resource, teacher autonomy exerts a stable driving force on teaching enthusiasm. Teachers with high autonomy internalize teaching goals and activities as self-determined behaviors, experiencing higher levels of intrinsic motivation and positive emotions in their teaching (Wang et al., 2025). This psychological resource, derived from autonomy support, exhibits cross-situational stability, sustaining teachers' teaching enthusiasm even in the absence of immediate external rewards or when facing teaching challenges (Moè and Katz, 2022). Empirically, Moè and Katz (2022) demonstrated that teacher autonomy functions as a key antecedent that fuels teaching enthusiasm through the mediation of intrinsic motivation. When teachers perceive that they have the freedom to control classroom instruction, teaching ceases to be the mechanical execution of external directives. Instead, it becomes a pathway to expressing their professional identity and realizing innovative value, thereby fostering deep and enduring teaching enthusiasm (Parker, 2015).

Furthermore, regarding the mediating role of teacher autonomy, existing research has provided relatively consistent empirical evidence. For example, Liu et al. (2021), based on a sample of Chinese teachers, found that teacher autonomy mediated the relationship between distributed leadership and job satisfaction. Lin's (2022) study showed that teacher autonomy mediated the relationship between distributed leadership and teacher innovativeness. Based on this, the following hypothesis is proposed:

**H2a: Distributed leadership is significantly positively correlated with teacher autonomy**.
**H2b: Teacher autonomy mediates the relationship between distributed leadership and teaching enthusiasm**.

### 2.5 The mediating role of organizational commitment

Organizational commitment is defined as a psychological state that reflects the relative strength of the bond between an employee and the organization and influences the employee's willingness to maintain ties with the organization (Meyer and Allen, 1991). As a multidimensional psychological construct, organizational commitment reflects teachers' attitudes or orientations toward the organization. Recent studies indicate that organizational commitment is a key factor in promoting work productivity and satisfaction (Erlangga et al., 2021) and enhancing organizational citizenship behavior (Bakhshi et al., 2011).

Distributed leadership promotes organizational commitment. Through power sharing and the cultivation of a collaborative culture, distributed leadership conveys to teachers the school's recognition of their professional value and respect for their contributions (Berjaoui and Karami-Akkary, 2020). This recognition and respect form the foundation of a high-quality social exchange relationship, enabling teachers to perceive the school not merely as a place of employment, but as a professional community worthy of emotional investment (Hulpia et al., 2009). Based on the resource investment hypothesis of COR theory, when teachers receive resources such as support, trust, and opportunities for participation from the organization, they tend to reciprocate with organizational commitment, creating a virtuous cycle of resource appreciation (Hulpia and Devos, 2010). This relationship has been confirmed in large-scale empirical studies. For example, Liu and Werblow (2019), in their multi-level analysis of TALIS 2013 data, found a significant positive relationship between distributed leadership and teachers' organizational commitment across multiple countries.

As a personal resource, organizational commitment have a positive role to drive teaching enthusiasm stably, Teachers with high emotional commitment internalize the school's values and goals as part of their self-concept, viewing teaching as a mission rather than merely a means of livelihood (Yan et al., 2023). This psychological resource, rooted in organizational identity, exhibits cross-situational stability and can sustain teachers' work engagement and teaching enthusiasm even in the absence of immediate external incentives or when facing teaching difficulties (Keller et al., 2013). At the same time, when teachers have a strong emotional attachment to their school, classroom teaching becomes not merely a task to be completed, but a means of expressing organizational identity and realizing self-worth, thereby leading to higher levels of teaching enthusiasm (Chen, 2007). This link is empirically supported by studies such as Chen (2007), who demonstrated that organizational commitment contributes to teachers' work enthusiasm by fostering a sense of belonging and mission. Similarly, Keller et al. (2013) noted that teachers' organizational commitment can sustain work engagement and enthusiasm even in the absence of immediate external incentives.

Furthermore, the mediating role of organizational commitment between leadership behaviors and teacher outcomes has received considerable empirical support. For example, Yao and Ma (2024), based on a sample of Chinese teachers, confirmed that organizational commitment mediated the relationship between distributed leadership and teacher career satisfaction. Bellibaş et al. (2024) found that teacher organizational commitment played a mediating role between distributed leadership and teacher turnover intention. In the relationship between leadership and teacher affective–motivational outcomes, organizational commitment has been repeatedly demonstrated as a key mediating pathway. Chen (2007) confirmed among Chinese secondary school teachers that organizational commitment mediated the relationship between job characteristics and teaching enthusiasm. Based on this, the following hypothesis is proposed:

**H3a: Distributed leadership is significantly and positively correlated with teachers' organizational commitment**.
**H3b: Organizational commitment mediates the relationship between distributed leadership and teaching enthusiasm**.

### 2.6 The chain mediation effect of teacher autonomy and organizational commitment

COR theory emphasizes that resource flow and resource transformation are key mechanisms for understanding resource associations. Halbesleben et al. (2014) point out that there are transmission and transformation pathways between different categories of resources: condition resources can create conditions for the formation of personal characteristic resources, and various resources within the resource cluster mutually support and derive from one another. Hobfoll et al. (2018) further elaborated on the “value-added spiral” effect of resource investment: the acquisition of one type of resource can promote the accumulation of other types of resources, forming a positive cycle of resource gains. This perspective on resource flow provides a theoretical foundation for understanding the relationship between teacher autonomy and organizational commitment.

Within the COR theoretical framework, teacher autonomy is defined as a condition resource that both promotes active resource investment and mitigates resource depletion, thereby playing a crucial role in enhancing teachers' organizational commitment. Specifically, greater autonomy grants teachers more discretion and a sense of responsibility, enabling them to make independent, flexible decisions about teaching styles and workflows, thereby fulfilling their psychological need for autonomy (Song and Hsieh, 2026). This not only significantly enhances job satisfaction but also effectively alleviates negative emotions such as emotional exhaustion, thereby promoting higher levels of teacher organizational commitment (Wang et al., 2026). Multiple recent empirical studies provide direct support for this inference. For example, Wang et al. (2026) found a significant positive correlation between teaching autonomy and Chinese teachers‘ organizational commitment, with self-efficacy and professional wellbeing serving as mediators. Similarly, Huang et al. (2021) demonstrated that university faculty's psychological empowerment, a construct closely related to autonomy, significantly predicts their organizational commitment. Therefore, based on the above analysis, the following hypothesis is proposed:

**H4a: Teacher autonomy is significantly positively correlated with teachers' organizational commitment**.
**H4b: Teacher Autonomy mediates the relationship between distributed leadership and teaching enthusiasm through organizational commitment**.

**Figure 1:**
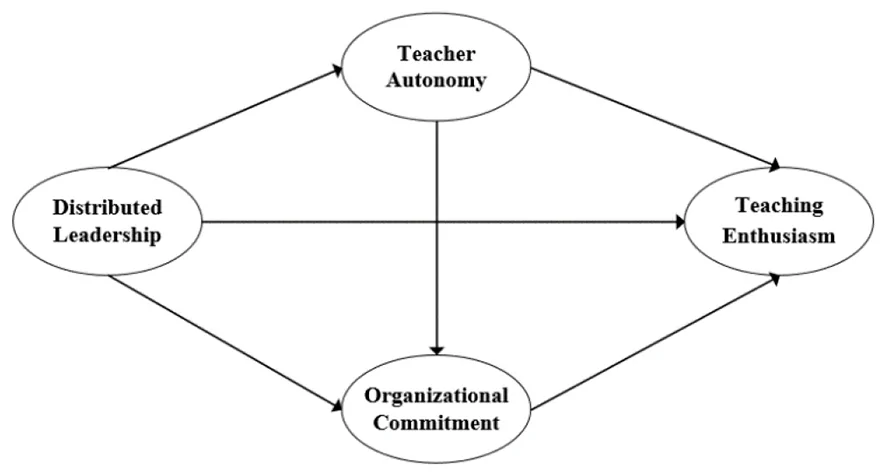
Research framework.

Based on the derivation of the above hypotheses, the research framework proposed in this study is shown in Figure 1.

## 3 Research methods

### 3.1 Data sources and sample characteristics

The data for this study are drawn from the fourth round of the Teaching and Learning International Survey (TALIS 2024), conducted by the Organization for Economic Co-operation and Development (OECD) in 2024. The TALIS project is one of the largest international surveys on teacher professionalism systematically collect comparable data on working conditions, professional development, and school leadership practices among lower-secondary school teachers in participating countries and regions, which is conducted approximately every 6 years. TALIS 2024 employed a stratified two-stage random sampling design: in the first stage, at least 200 lower-secondary schools were randomly selected from each participating country or region; in the second stage, approximately 20 teachers were randomly selected from each sampled school. This study focuses on the teacher sample from Shanghai, China. Shanghai first participated independently in TALIS 2018 in 2019, and its teachers' performance attracted widespread attention, then its continued participation in the 2024 survey provides high-quality, temporally continuous data for research.

TALIS 2024 employs a cyclical survey design; consequently, there are design-related missing values when items are presented to only a portion of the sample (1/3 or 2/3) or to the entire sample. Since such missing values are considered completely random (MCAR) (Organisation for Economic Co-operation Development, 2025). To prevent bias in parameter estimates and maintain sample consistency during the analysis, this study utilized cases with no missing data on the primary variable measurement items. Ultimately, a valid analytical sample of 1,319 teachers was obtained. The demographic characteristics of the sample are as shown in Table 1. In terms of gender distribution, there were 962 female teachers (72.93%) and 357 male teachers (27.07%). In terms of educational background, the vast majority of teachers held a bachelor's degree, totaling 1,021 (77.41%); 292 teachers (22.14%) held a master's or doctoral degree; and only 6 teachers (0.45%) had an educational level below a bachelor's degree, indicating a high overall educational level within the sample. In terms of years of teaching experience, the largest group consists of senior teachers with over 20 years of experience, totaling 483 (36.62%); 349 teachers (26.46%) have 11–20 years of experience; 226 teachers (17.13%) have 6–10 years of experience; and 261 novice teachers (19.79%) have 5 years or less of experience.

**Table 1:** Demographic characteristics of the sample (*N* = 1,319).

Variable	Category	*N*	Percentage (%)
Gender	Female	962	72.93
	Male	357	27.07
Education background	Less than a bachelor's degree	6	0.45
	Bachelor's degree	1,021	77.41
	Graduate degree	292	22.14
Years of teaching experience	5 years or less	261	19.79
	6–10 years	226	17.13
	11–20 years	349	26.46
	Over 20 years	483	36.62

### 3.2 Measurement

All core variables in this study were measured using established scales from the TALIS 2024 Teacher Questionnaire. All items were scored on a 4-point Likert scale. These scales have undergone repeated cross-cultural pilot testing and psychometric validation across multiple rounds of the TALIS survey, which is well-established evidence of reliability and validity. In the present study, the Cronbach's alpha coefficients for each scale exceeded the recommended threshold of 0.70.

#### 3.2.1 Distributed leadership

Distributed leadership was measured using the Perceived Distributed Leadership Scale from the teacher questionnaire. Consistent with the operationalization in prior research using TALIS data (e.g., Liu et al., 2021; Bellibaş et al., 2024). This scale consists of five items and measures the extent to which teachers perceive their principal to practice distributed leadership in school management. A typical item is: “This school provides staff with opportunities to actively participate in school decisions.” The scale uses a 4-point rating scale (1 = Strongly Disagree, 4 = Strongly Agree), with the mean score of all items serving as the indicator of distributed leadership; a higher score indicates a stronger degree of distributed leadership as perceived by teachers. In this study, the Cronbach's alpha coefficient for this scale was 0.954. The results of confirmatory factor analysis indicated acceptable construct validity: RMSEA = 0.027, CFI = 0.998, TLI = 0.994, and SRMR = 0.005. The standardized factor loadings ranged from 0.819 to 0.932. All major fit indices met the recommended standards, indicating that the scale can effectively measure the level of distributed leadership.

#### 3.2.2 Teacher autonomy

Teacher Autonomy was measured using the “Teacher Autonomy Scale” in the teacher questionnaire. Specifically, teachers were asked: To what extent do you have control over the following areas of your planning and teaching in the target class? such as “determining course content” “selecting teaching methods” “assessing students' learning,” “disciplining students,” and “determining the amount of homework to be assigned.” A four-point Likert scale was used, with scores ranging from “1 = Strongly Disagree” to “4 = Strongly Agree”; higher scores indicate a higher level of Teacher Autonomy. Research by Wang and Bai (2025) indicates that these items effectively assess Teacher Autonomy and possess good reliability and validity. In this study, the Cronbach's alpha coefficient for the scale was 0.914. The results of confirmatory factor analysis indicated acceptable construct validity: RMSEA = 0.052, CFI = 0.992, TLI = 0.979, and SRMR = 0.014. The standardized factor loadings ranged from 0.798 to 0.896. All major fit indices met the recommended standards, indicating that the scale can effectively measure the level of teacher autonomy.

#### 3.2.3 Organizational commitment

According to the studies by Bellibaş et al. (2024) and Wang et al. (2026), organizational commitment was measured using three items in the teacher questionnaire, including “I would like to change to another school if that were possible (reverse-scored),” “I enjoy working at this school,” and “I would recommend this school as a good place to work.” A four-point Likert scale was used, with responses ranging from “1 = Strongly Disagree” to “4 = Strongly Agree”; higher scores indicate stronger organizational commitment among teachers. In this study, the Cronbach's alpha coefficient for this scale was 0.726. Since the scale contains only three items, the confirmatory factor analysis model is just-identified (saturated) with zero degrees of freedom; therefore, conventional model fit indices (e.g., RMSEA, CFI, and TLI) cannot be meaningfully interpreted. Consequently, construct validity was assessed solely through composite reliability (CR) and average variance extracted (AVE). As reported in Table 2, the CR value was 0.753, exceeding the 0.70 threshold, and the AVE was 0.516, above the 0.50 criterion, indicating acceptable convergent validity. The standardized factor loadings ranged from 0.490 to 0.810. Taken together, the scale demonstrates adequate reliability and validity for measuring teachers' organizational commitment. However, it should be noted that this scale measures organizational commitment as a global construct and primarily captures teachers' affective attachment to the school. It does not differentiate among the affective, continuance, and normative dimensions of commitment as proposed by Meyer and Allen (1991). This unidimensional operationalization limits the ability to examine potentially distinct relationships that each commitment dimension may exhibit with distributed leadership and teaching enthusiasm.

**Table 2:** Reliability and validity.

Variables	Cronbach's α	CR	AVE	HTMT			
				1	2	3	4
1. Distributed leadership	0.954	0.953	0.804	1			
2. Teacher autonomy	0.914	0.921	0.701	0.348	1		
3. Organizational commitment	0.726	0.753	0.516	0.596	0.296	1	
4. Teaching enthusiasm	0.913	0.914	0.726	0.410	0.328	0.558	1

#### 3.2.4 Teaching enthusiasm

Teaching enthusiasm was measured using the new teacher teaching enthusiasm scale introduced in TALIS 2024. This scale consists of four items, such as “I like the subject(s) that I teach” and “I often feel happy while I teach.” The scale uses a 4-point rating scale (1 = Strongly Disagree, 4 = Strongly Agree), with the mean of all items serving as the indicator of teaching enthusiasm; a higher score indicates greater teaching enthusiasm. Although teaching enthusiasm has been theorized as a multidimensional construct comprising enthusiasm for teaching and enthusiasm for the subject matter (Kunter, 2013; Keller et al., 2016), the TALIS 2024 scale was designed to assess teachers' overall teaching enthusiasm as a global, unidimensional indicator. The four items capture enjoyment of the subject taught, happiness while teaching, the expression of enthusiasm, and satisfaction derived from teaching challenges, thus representing a general affective–motivational engagement in teaching. Treating the scale as unidimensional aligns with the TALIS 2024 conceptual framework (Organisation for Economic Co-operation Development, 2025) and is supported by the high internal consistency and the excellent fit of a single-factor model. This approach is consistent with recent empirical studies (e.g., Doguş, 2026). Nevertheless, we acknowledge that a unidimensional operationalization may not differentiate between enthusiasm for the subject and enthusiasm for the act of teaching, a limitation addressed in the discussion of future research directions. In this study, the Cronbach's alpha coefficient for this scale was 0.913. The results of confirmatory factor analysis indicated acceptable construct validity: RMSEA = 0.016, CFI = 0.999, TLI = 0.998, and SRMR = 0.006. The standardized factor loadings ranged from 0.808 to 0.885. All major fit indices met the recommended standards, indicating that the scale can effectively measure teachers' levels of teaching enthusiasm.

This study included teachers' gender (female = 0, male = 1), years of teaching experience (continuous variable), highest level of education (continuous variable), whether teaching was their first career choice (yes = 1, no = 0), and school location (urban = 1, rural = 0) as control variables in the model. Controlling for these variables helps reduce the confounding effects of individual and school characteristics on the relationship between the core variables and the outcome.

### 3.3 Data analysis

Data analysis was conducted using Stata 17 and Mplus 8.3 software. The first step involved common method bias testing and descriptive statistical analysis. The second step involved structural equation modeling and hypothesis testing. Structural equation models were constructed using Mplus 8.3 with the MLR (Maximum Likelihood with Robust Standard Errors) estimator. Given the large sample size, the common practice of treating Likert-scale items as continuous variables in TALIS research, and the necessity to account for clustering and sampling weights, MLR was chosen because it provides robust standard errors and is robust to non-normality. The direct association between distributed leadership and teaching enthusiasm was estimated, as well as the indirect relationships through teacher autonomy and organizational commitment. Third, the bias-corrected percentile bootstrap method (with 5,000 repetitions) was employed to test the significance of the mediating relationships. The bootstrap method does not rely on the assumption that indirect associations follow a normal distribution and offers clear statistical advantages when dealing with mediating relationship estimates that often exhibit skewed distributions. When using Mplus for parameter estimation and standard error calculation, the study employed teacher weights (TCHWGT) and school identifiers (IDSCHOOL) from the TALIS dataset as weighting and clustering variables to address unequal sampling probabilities and non-independence among teachers (Özdemir et al., 2023).

## 4 Research results

### 4.1 Common method bias test

All variables in this study were collected via a one-time questionnaire completed by teachers, which may introduce common method bias. The Harman single-factor test was used to assess this issue. All items from the four scales include distributive leadership, teacher autonomy, organizational commitment, and teacher teaching enthusiasm, were included in exploratory factor analysis (unrotated). The results showed that there were four factors with eigenvalues greater than 1, and the first common factor explained 30.17% of the total variance, which is below the critical threshold of 40% (Podsakoff et al., 2012). This result indicates that there is no serious common method bias in this study. However, acknowledging the limited sensitivity of the Harman test, this study additionally performed a single-factor confirmatory factor analysis (CFA). The results demonstrated a poor model fit (RMSEA = 0.178, CFI = 0.499, TLI = 0.427, SRMR = 0.188), further confirming that the common method bias is not serious.

### 4.2 Reliability and validity

As shown in Table 2, the Cronbach's α coefficients for Distributed Leadership, Teacher Autonomy, Organizational Commitment, and Teaching Enthusiasm were 0.954, 0.914, 0.726, and 0.913, respectively. The composite reliability (CR) values were 0.953, 0.921, 0.753, and 0.914, respectively, all of which met the recommended threshold of 0.7, indicating that the measurement of each latent variable has good internal consistency. Regarding convergent validity, the average variance extracted (AVE) for the four variables was 0.804, 0.701, 0.516, and 0.726, respectively, all exceeding the critical threshold of 0.5. This indicates that the items for each construct explain more than 50% of the variance, demonstrating ideal convergent validity. Regarding discriminant validity, the Heterogeneous-to-Monolithic Ratio (HTMT) test results showed that all HTMT values were below the 0.85 threshold, supporting discriminant validity (Henseler et al., 2015). In summary, the reliability and validity of the measurement tools used in this study are at an ideal level and are suitable for subsequent structural equation modeling analysis.

### 4.3 Descriptive statistics and correlation analysis

Table 3 presents the means, standard deviations, skewness, kurtosis and Pearson correlation coefficient matrix for each core variable. The skewness values for all variables ranged from −0.472 to −0.059, and kurtosis values ranged from 2.333 to 3.488, indicating that the data distributions were approximately normal and met the assumptions for parametric analyses. Distributed leadership was significantly positively correlated with teaching enthusiasm (*r* = 0.385, *p* < 0.001), with a moderate effect size. Distributed leadership was significantly positively correlated with teacher autonomy (*r* = 0.323, *p* < 0.001) and with organizational commitment (*r* = 0.504, *p* < 0.001). Teacher autonomy was significantly positively correlated with teaching enthusiasm (*r* = 0.299, *p* < 0.001) and with organizational commitment (*r* = 0.250, *p* < 0.001). Organizational commitment was significantly positively correlated with teaching enthusiasm (*r* = 0.461, *p* < 0.001). Furthermore, none of the correlation coefficients between variables exceeded the multicollinearity threshold of 0.70, and all variance inflation factors were below 3.0, indicating no serious multicollinearity.

**Table 3:** Descriptive statistics and correlation analysis.

Variables	M	SD	Skewness	Kurtosis	1	2	3	4
1. Distributed leadership	3.283	0.591	−0.472	3.488	1			
2. Teacher autonomy	3.185	0.590	−0.138	2.333	0.323^***^	1		
3. Organizational commitment	2.972	0.579	−0.128	3.163	0.504^***^	0.250^***^	1	
4. Teaching enthusiasm	3.298	0.523	−0.059	2.732	0.385^***^	0.299^***^	0.461^***^	1

^***^p < 0.001.

M, mean; SD, standard deviation.

### 4.4 Structural equation modeling and hypothesis testing

The results of the structural equation modeling analysis indicate that the theoretical model fits the empirical data well. The model fit indices are as follows: RMSEA = 0.044, CFI = 0.957, TLI = 0.951, and SRMR = 0.043. All indicators reached excellent levels, indicating that the theoretical model effectively reflects the structural relationships among the variables.

To establish the robustness of the proposed chain mediation model, we compared it with a competing parallel mediation model (i.e., removing the path from Teacher Autonomy to Organizational Commitment, allowing both mediators to influence Teaching Enthusiasm in parallel). The comparison of model fit revealed that the chi-square difference (Δχ^2^ = 214.205, Δ*df* = 1) was statistically significant (*p* < 0.001), indicating that the hypothesized chain mediation model is significantly superior to the competing parallel mediation model. Furthermore, the fit indices of the hypothesized model were all superior to those of the competing model (RMSEA = 0.045, CFI = 0.926, TLI = 0.910, SRMR = 0.086). Based on the results of the above comparison tests, the observed data fit best with the proposed model; therefore, the chain mediation model is accepted.

**Figure 2:**
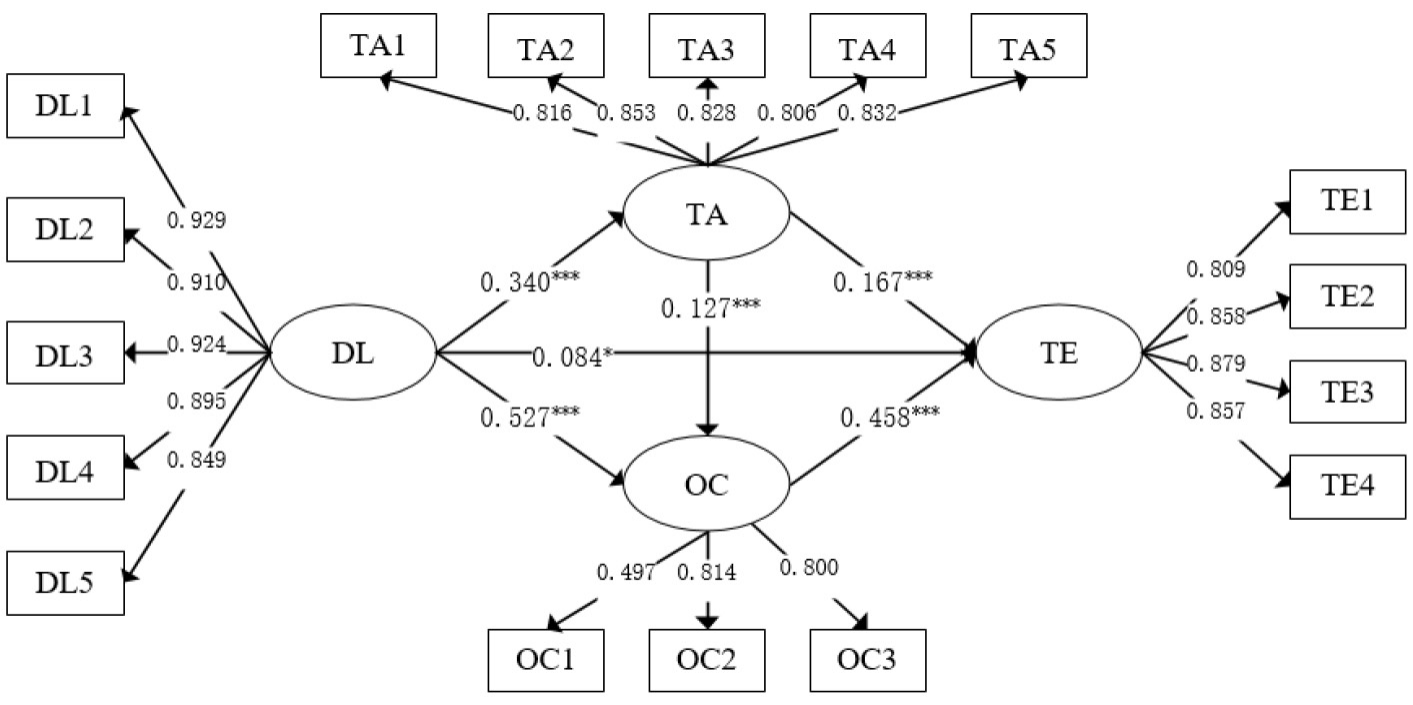
Path coefficients of the structural equation model. DL, Distributed Leadership; TA, Teacher Autonomy; OC, Organizational Commitment; TE, Teaching Enthusiasm.

The results of the structural equation modeling analysis are shown in Figure 2. The model explained 11.6% of the variance in teacher autonomy (*R*^2^ = 0.116), 33.9% of the variance in organizational commitment (*R*^2^ = 0.339), and 35.5% of the variance in teaching enthusiasm (*R*^2^ = 0.355). Significant positive correlations exist among all study variables. Distributed leadership is significantly positively correlated with teaching enthusiasm (*β* = 0.084, *p* < 0.05). Research hypothesis H1 is supported. Distributed leadership is significantly and positively correlated with teacher autonomy (*β* = 0.340, *p* < 0.001) and with organizational commitment (*β* = 0.527, *p* < 0.001). Teacher autonomy is significantly and positively correlated with organizational commitment (*β* = 0.127, *p* < 0.001) and with teaching enthusiasm (*β* = 0.167, *p* < 0.001). There is also a significant positive correlation between organizational commitment and teaching enthusiasm (*β* = 0.458*, p* < 0.001). All of the above correlation coefficients were highly significant, indicating a strong positive association among the variables and providing the necessary conditions for subsequent tests of mediation effects. The corresponding hypotheses are supported. Among the control variables, years of teaching experience was significantly negatively associated with teaching enthusiasm (*β* = −0.081, *p* = 0.002). Gender (*β* = −0.002, *p* = 0.947), educational level (*β* = 0.037, *p* = 0.169), Teaching as a first career choice (*β* = 0.017, *p* = 0.577), and school location (*β* = −0.003, *p* = 0.885) did not show significant associations with teaching enthusiasm.

The study employed a bias-corrected percentile bootstrap method (5,000 bootstrap replicates) to test each mediating pathway rigorously. As shown in Table 4, the total association of distributed leadership on teaching enthusiasm was 0.402 (SE = 0.031, 95% CI [0.340, 0.463]), representing a medium-to-large effect size (Cohen, 1988). The direct association was 0.084 (SE = 0.040, 95% CI [0.005, 0.163]), which is a small but statistically significant effect, indicating that distributed leadership exerts a modest direct influence on teaching enthusiasm beyond indirect pathways. The total indirect association was 0.318 (SE = 0.028, 95% CI [0.263, 0.373]), representing a medium effect size.

**Table 4:** Test of mediating effect.

Path	Effect	SE	95% CI		Proportion (%)
			Lower	Upper	
Total effect	0.402	0 0.031	0.340	0.463	
Direct effect	0.084	0.040	0.005	0.163	20.90
Indirect effect	0.318	0.028	0.263	0.373	79.10
DL → TA → TE	0.057	0.012	0.034	0.080	14.18
DL → OC → TE	0.241	0.024	0.194	0.288	59.95
DL → TA → OC → TE	0.020	0.006	0.009	0.031	4.98

DL, Distributed Leadership; TA, Teacher Autonomy; OC, Organizational Commitment; TE, Teaching Enthusiasm.

The sum of the direct effect percentages is 100.01%; the sum of the indirect effect percentage and the three indirect path percentages is 79.11%. Minor discrepancies are due to rounding.

The specific indirect relationship of Teacher Autonomy (DL → TA → TE) was 0.057, with a standard error of 0.012 and a 95% confidence interval of [0.034, 0.080], which does not include zero, indicating that Teacher Autonomy plays a significant independent mediating role in the process by which distributed leadership influences teaching enthusiasm, representing that H2b is supported. This indirect effect corresponds to a small effect size, consistent with autonomy serving as a modest but significant transmission pathway. The specific indirect relationship of Organizational Commitment (DL → OC → TE) was 0.241, with a standard error of 0.024 and a 95% confidence interval of [0.194, 0.288], which does not include zero, confirming that Organizational Commitment also plays a significant independent mediating role, representing that H3b is supported. This effect is moderate in size, underscoring the pivotal role of organizational commitment as the dominant mediating pathway. The chain mediating relationship of Teacher Autonomy and Organizational Commitment (DL → TA → OC → TE) yielded a value of 0.020, with a standard error of 0.006 and a 95% confidence interval of [0.009, 0.031], which does not include zero. This indicates that the two variables constitute a significant chain mediation path, and H4b is supported. Although this chain effect is small in magnitude, which is theoretically expected given the multiplicative nature of sequential mediation, its statistical significance provides empirical support for the resource flow and value-added spiral assumptions of COR theory.

## 5 Discussion

First, distributed leadership is significantly positively correlated with teachers' teaching enthusiasm. This result is consistent with the broader evidence base showing that distributed leadership fosters positive teacher outcomes such as wellbeing and engagement (Doguş, 2026; Buyukgoze et al., 2024), and it directly aligns with recent work by Doguş (2026), who found that distributed leadership is positively related to teachers' enthusiasm in Türkiye. Thus, our study replicates and reinforces this association within the distinct cultural and institutional setting of Shanghai, China. From the perspective of COR theory, distributed leadership serves as a resource channel at the organizational level. Through empowerment practices and a collaborative culture, it provides teachers with opportunities for decision-making participation and peer support. These resource gains are transformed into the psychological energy that sustains teaching enthusiasm. This finding underscores that distributed leadership not only promotes general teacher wellbeing but specifically ignites the passion for teaching itself, an outcome that is pivotal for instructional quality and inherently distinct from broad affective states such as job satisfaction or global engagement. In the local context, although China's educational management system has long maintained a hierarchical tradition, the empowering function of distributed leadership can still serve as a resource-supplying role within this framework, providing teachers with psychological support that transcends structural constraints.

Second, Teacher Autonomy serves as an independent mediator between distributed leadership and teaching enthusiasm. the identified mediating role of teacher autonomy is highly consistent with prior studies that have demonstrated autonomy as a key mediating mechanism between distributed leadership and teacher outcomes such as job satisfaction (Liu et al., 2021), innovativeness (Lin, 2022; Gong, 2025), and well-being (Song and Hsieh, 2026). This pathway reveals the resource-transmission logic by which distributed leadership is indirectly related to teaching enthusiasm by empowering teachers' decision-making space in teaching. The core practices of distributed leadership are power sharing and empowerment, and teacher autonomy is the most direct manifestation of empowerment in school teaching contexts. When teachers possess substantial discretion in curriculum design, method selection, and assessment strategies, their teaching behavior shifts from passive execution to active creation. This sense of control, rooted in the need for autonomy, is a key component of intrinsic motivation and serves as the psychological fuel that sustains teaching enthusiasm. Notably, distributed leadership explained a modest proportion of variance in teacher autonom, indicating that a considerable portion of teachers' perceived autonomy is shaped by factors beyond empowerment and collaborative structures. Potential unmeasured antecedents include organizational characteristics such as school climate, administrative trust, and curriculum prescription, as well as individual psychological resources like professional competence, teacher self-efficacy, and career stage. The modest explained variance does not diminish the theoretical importance of the identified pathway, but it calls for future research to incorporate a broader set of predictors and to examine how distributed leadership interacts with these factors in shaping teacher autonomy.

Third, organizational commitment serves as an independent mediator between distributed leadership and teaching enthusiasm, and this pathway exhibits the largest effect size among the three conditional mediating pathways. This finding aligns with and extends the work of Yao and Ma (2024), who reported that organizational commitment mediates the relationship between distributed leadership and career satisfaction among Chinese teachers. Similarly, our results corroborate Bellibaş et al. (2024), who found that organizational commitment serves as a mediator linking distributed leadership to teacher turnover intentions. This result highlights the pivotal role of the emotional bond between teachers and their schools in the process of empowering distributed leadership. By fostering an organizational atmosphere of trust, cooperation, and shared responsibility, distributed leadership catalyzes teachers' emotional identification with and sense of belonging to the school. Once established, organizational commitment becomes a stable personal resource that drives teachers to transcend the logic of instrumental exchange, internalizing teaching as mission-driven work and thereby enhancing their teaching enthusiasm.

Fourth, the chain mediation effect between Teacher Autonomy and organizational commitment is significant. This depicts a clear sequence of resource transmission: distributed leadership → Teacher Autonomy (condition resource) → organizational commitment (personal characteristic resource) → teaching enthusiasm (positive emotional state). Distributed leadership first provides teachers with autonomy through empowerment; teachers then transform this institutional experience of autonomy into emotional commitment to the school through psychological processes such as attribution and trust signals. The convergence of these two resources jointly drives teaching enthusiasm. This finding empirically validates the core assumptions of the resource flow and value-added spiral in COR theory (Hobfoll et al., 2018), overcoming the limitations of previous research that treated mediating variables as parallel, isolated mechanisms, and elucidates the sequential psychological processes through which distributed leadership empowers teachers.

Nevertheless, the practical significance of this sequential mediation pathway should be interpreted with caution. Although statistically significant, the chain indirect effect is relatively small. This modest magnitude suggests that the incremental value generated by transforming autonomy into commitment, while theoretically meaningful, may have a limited standalone practical impact on enhancing teaching enthusiasm. However, this does not negate its practical importance; rather, it indicates that fostering teaching enthusiasm through distributed leadership likely requires a holistic approach that simultaneously activates multiple resource channels, rather than relying solely on the sequential transmission from autonomy to commitment. Furthermore, the small effect size is consistent with the inherent nature of chain mediation, where the product of multiple path coefficients typically yields a numerically modest indirect effect. Thus, this pathway should be viewed as one component of a broader resource ecology, and its practical implications should be understood in conjunction with the other, stronger mediating mechanisms identified in this study.

## 6 Implications for practice

This study provides evidence-based practical insights for educational administrators, school leaders, and policymakers. Key findings of this study are as follows:

First, it needs to incorporate distributed leadership into principal training and evaluation systems. The study found that distributed leadership significantly enhances teaching enthusiasm through a chain mediation involving teachers' pedagogical autonomy and organizational commitment. Therefore, training should systematically include modules on empowerment theory, shared decision-making, and collaborative culture to encourage principals to shift from being “heroic leaders” to “leadership cultivators.” Training content should cover identifying strengths, democratic decision-making, team building, and techniques for balancing empowerment, and distributed leadership behaviors should be incorporated into principal performance evaluations. In line with China's “Principal Responsibility System” and the national strategy for building a high-quality teaching force, these findings suggest that principal evaluation criteria should include distributed leadership indicators, and that training programs should draw on case studies from exemplary Chinese schools that have successfully democratized decision-making, thus providing localized models for leadership development.

Second, it needs to establish institutional mechanisms to safeguard teaching autonomy. Teaching autonomy serves as the activating mediator through which distributed leadership influences teaching enthusiasm; its absence disrupts the transmission chain. Schools must institutionally guarantee teachers' professional decision-making authority. For example, by establishing curriculum and teaching review committees with substantive deliberative powers, supporting teaching and research teams in leading educational reforms, and designing evaluation systems that reflect innovative autonomy, thereby making empowerment a stable institutional arrangement. Given China's strong tradition of school-based teaching research groups (jiaoyanzu), schools can leverage these existing collaborative structures to institutionalize teacher autonomy by empowering subject groups to co-determine curriculum content, assessment methods, and professional learning activities, thus bridging policy mandates with classroom realities and aligning with the new curriculum reform's emphasis on school-based implementation.

Third, it needs to cultivate organizational commitment as the strategic direction for school culture construction. Organizational commitment is the mediator with the greatest effect in this study; schools should strive to foster a high-commitment culture, transforming the school into a community of shared purpose through fair management, professional support, transparent communication, and a shared vision. Measures include participatory management to ensure informed decision-making and a voice, establishing mutual support networks to enhance professional interaction, and reinforcing shared values through rituals, narratives, and role models. Such efforts align with the national vision of cultivating a highly professional and dedicated teaching body and can be reinforced by integrating commitment-building practices into school quality monitoring frameworks under China's compulsory education standards, thereby translating the policy goal of “making teaching an enviable profession” into concrete school-level actions.

## 7 Research limitations and future directions

This study has the following limitations: First, the cross-sectional design limits causal inference, and reverse causality may exist among variables. Future research should adopt a longitudinal design, collecting panel data at least three time points and using cross-lagged panel models to test causal sequences. Second, all variables are self-reported by teachers; although common-method bias is acceptable, social desirability and subjective bias still exist. Future research should integrate multi-source data (principal self-assessments, peer evaluations, classroom observations) and incorporate qualitative methods (in-depth interviews, case studies). Third, Future research on the relationship between distributed leadership and teaching enthusiasm, grounded in COR theory, should expand mediating mechanisms by systematically examining teacher self-efficacy, psychological capital, school climate, principal support, job satisfaction, and professional identity as parallel mediators, alternative resources, or moderators. Although the present study's exclusive focus on teacher autonomy and organizational commitment aligns with COR theory's emphasis on condition and personal-characteristic resources, a single study cannot capture all pathways—future work should integrate these broader psychological and organizational variables to build more comprehensive models and test the boundary conditions and incremental explanatory power of each pathway. Fourth, organizational commitment was operationalized as a unidimensional global construct. Although the three-item scale demonstrated acceptable reliability and validity for measuring overall commitment, it does not distinguish between affective, continuance, and normative commitment. According to COR theory, different commitment types may function as distinct resource categories and could exhibit different patterns of association with distributed leadership and teaching enthusiasm. For example, affective commitment, rooted in emotional attachment, might be more strongly linked to intrinsic teaching enthusiasm, whereas continuance commitment, based on perceived costs of leaving, could relate to a more calculative, less passionate engagement. Future studies should adopt multidimensional commitment scales (e.g., Meyer and Allen's three-component model) to disentangle these differential effects and examine whether the mediating roles identified here vary across commitment dimensions. Fifth, the study relied on the unidimensional TALIS 2024 teaching enthusiasm scale. While this is consistent with the survey design and prior research, it may mask potentially distinct pathways through which distributed leadership influences enthusiasm for the subject vs. enthusiasm for the act of teaching. Future research should employ multidimensional measures to disentangle these differential effects and examine whether autonomy and commitment play different roles for each enthusiasm dimension. Sixth, as the sample is limited to junior high schools in Shanghai, caution is warranted when generalizing the findings to other regions, educational levels, and cultures. Shanghai's educational development is advanced, and its management practices and teachers' psychological profiles may differ from those in central and western regions and rural areas. Future research could utilize the TALIS 2024 cross-national comparison or collect targeted regional samples.

## Data Availability

Publicly available datasets were analyzed in this study. This data can be found here: The data analyzed in this study are publicly available from the TALIS 2024 Data Repository. The data can be accessed at: https://www.oecd.org/en/data/datasets/talis-2024-database.html.

## References

[B1] Bakhshi A. Sharma A. D. Kumar K. (2011). Organizational commitment as a predictor of organizational citizenship behavior. Eur. J. Bus. Manag., 3, 78–86.

[B2] Bellibaş M. S. Gümüş S. Chen J. (2024). The impact of distributed leadership on teacher commitment: the mediating role of teacher workload stress and teacher well-being. Br. Educ. Res. J. 50, 814–836. doi: 10.1002/berj.3944

[B3] Berjaoui R. R. Karami-Akkary R. (2020). Distributed leadership as a path to organizational commitment: the case of a Lebanese school. Leadersh. Policy Schools 19, 610–624. doi: 10.1080/15700763.2019.1637900

[B4] Buyukgoze H. Caliskan O. Gümüş S. (2024). Linking distributed leadership with collective teacher innovativeness: the mediating roles of job satisfaction and professional collaboration. Educ. Manag. Admin. Leadersh. 52, 1388–1409. doi: 10.1177/17411432221130879

[B5] Chen W. (2007). The structure of secondary school teacher job satisfaction and its relationship with attrition and work enthusiasm. Chin. Educ. Soc. 40, 17–31. doi: 10.2753/CED1061-1932400503

[B6] Cohen J. (1988). Statistical Power Analysis for the Behavioral Sciences. London: Routledge Academic.

[B7] Collie R. J. Shapka J. D. Perry N. E. Martin A. J. (2016). Teachers' psychological functioning in the workplace: exploring the roles of contextual beliefs, need satisfaction, and personal characteristics. J. Educ. Psychol. 108, 788–799. doi: 10.1037/edu0000088

[B8] Doguş Y. (2026). The relationship between distributed leadership and teachers' enthusiasm and professional collaboration levels in Türkiye: the moderating effect of teacher optimism. Front. Psychol. 17:1729734. doi: 10.3389/fpsyg.2026.1729734 41858449 PMC12996238

[B9] Erlangga H. Mulyana Y. Sunarsi D. Solahudin M. Dwiwarman D. A. Waskita N. I. D. . (2021). The effect of organizational commitment and work environment on job satisfaction and teachers' performance. Turk. J. Comput. Math. Educ. 12, 109–117.

[B10] Frenzel A. C. Becker-Kurz B. Pekrun R. Goetz T. Lüdtke O. (2018). Emotion transmission in the classroom revisited: a reciprocal effects model of teacher and student enjoyment. J. Educ. Psychol. 110, 628–648. doi: 10.1037/edu0000228

[B11] Frenzel A. C. Taxer J. L. Schwab C. Kuhbandner C. (2019). Independent and joint effects of teacher enthusiasm and motivation on student motivation and experiences: a field experiment. Motiv. Emot. 43, 255–265. doi: 10.1007/s11031-018-9738-7

[B12] Gong J. (2025). The effects of distributed leadership on teaching innovation in Shanghai, China: the mediating roles of teacher autonomy, teacher collaboration, and teacher self-efficacy. Front. Psychol. 16:1562838. doi: 10.3389/fpsyg.2025.1562838 40727058 PMC12301307

[B13] Gronn P. (2002). Distributed leadership as a unit of analysis. Leadersh. Quart. 13, 423–451. doi: 10.1016/S1048-9843(02)00120-0

[B14] Ha C. Pressley T. Marshall D. T. (2025). Teacher voices matter: the role of teacher autonomy in enhancing job satisfaction and mitigating burnout. PLoS ONE 20:e0317471. doi: 10.1371/journal.pone.0317471 39804894 PMC11729918

[B15] Halbesleben J. R. Neveu J. P. Paustian-Underdahl S. C. Westman M. (2014). Getting to the “COR”: understanding the role of resources in conservation of resources theory. J. Manage. 40, 1334–1364. doi: 10.1177/0149206314527130

[B16] Harris A. (2008). Distributed leadership: according to the evidence. J. Educ. Admin. 46, 172–188. doi: 10.1108/09578230810863253

[B17] Hawkins J. N. (2000). Centralization, decentralization, recentralization: educational reform in China. J. Educ. Admin. 38, 442–455. doi: 10.1108/09578230010378340

[B18] Henseler J. Ringle C. M. Sarstedt M. (2015). A new criterion for assessing discriminant validity in variance-based structural equation modeling. J. Acad. Market. Sci. 43, 115–135. doi: 10.1007/s11747-014-0403-8

[B19] Hobfoll S. E. (1989). Conservation of resources: a new attempt at conceptualizing stress. Am. Psychol. 44, 513–524. doi: 10.1037/0003-066X.44.3.513 2648906

[B20] Hobfoll S. E. (2011). Conservation of resource caravans and engaged settings. J. Occup. Organ. Psychol. 84, 116–122. doi: 10.1111/j.2044-8325.2010.02016.x

[B21] Hobfoll S. E. Halbesleben J. Neveu J. P. Westman M. (2018). Conservation of resources in the organizational context: the reality of resources and their consequences. Annu. Rev. Organ. Psychol. Organ. Behav. 5, 103–128. doi: 10.1146/annurev-orgpsych-032117-104640

[B22] Huang Y. T. Liu H. Huang L. (2021). How transformational and contingent reward leaderships influence university faculty's organizational commitment: the mediating effect of psychological empowerment. Stud. High. Educ. 46, 2473–2490. doi: 10.1080/03075079.2020.1723534

[B23] Hulpia H. Devos G. (2010). How distributed leadership can make a difference in teachers' organizational commitment? a qualitative study. Teach. Teach. Educ. 26, 565–575. doi: 10.1016/j.tate.2009.08.006

[B24] Hulpia H. Devos G. Van Keer H. (2009). The influence of distributed leadership on teachers' organizational commitment: a multilevel approach. J. Educ. Res. 103, 40–52. doi: 10.1080/00220670903231201

[B25] Keller M. M. Goetz T. Becker E. S. Morger V. Hensley L. (2014). Feeling and showing: a new conceptualization of dispositional teacher enthusiasm and its relation to students' interest. Learn. Instr. 33, 29–38. doi: 10.1016/j.learninstruc.2014.03.001

[B26] Keller M. M. Hoy A. W. Goetz T. Frenzel A. C. (2016). Teacher enthusiasm: Reviewing and redefining a complex construct. Educ. Psychol. Rev. 28, 743–769. doi: 10.1007/s10648-015-9354-y

[B27] Keller M. M. Neumann K. Fischer H. E. (2013). “Teacher enthusiasm and student achievement,” in International Guide to Student Achievement, eds. J. Hattie, and E. M. Andermann (London: Routledge), 247–250.

[B28] Kilinç A. Ç. Polatcan M. Savaş G. Er E. (2024). How transformational leadership influences teachers' commitment and innovative practices: understanding the moderating role of trust in the principal. Educ. Manag. Admin. Leadersh. 52, 455–474. doi: 10.1177/17411432221082803

[B29] Kunter M. (2013). “Motivation as an aspect of professional competence: Research findings on teacher enthusiasm,” in Cognitive Activation in the Mathematics Classroom and Professional Competence of Teachers: Results From the COACTIV Project, eds. M. Kunter, J. Baumert, W. Blum, U. Klusmann, S. Krauss, and M. Neubrand (Boston, MA: Springer), 273–289. doi: 10.1007/978-1-4614-5149-5_13

[B30] Kunter M. Frenzel A. Nagy G. Baumert J. Pekrun R. (2011). Teacher enthusiasm: dimensionality and context specificity. Contemp. Educ. Psychol. 36, 289–301. doi: 10.1016/j.cedpsych.2011.07.001

[B31] Lin Q. (2022). The relationship between distributed leadership and teacher innovativeness: mediating roles of teacher autonomy and professional collaboration. Front. Psychol. 13:948152. doi: 10.3389/fpsyg.2022.948152 35967729 PMC9364974

[B32] Liu L. Hallinger P. (2018). Principal instructional leadership, teacher self-efficacy, and teacher professional learning in China: Testing a mediated-effects model. Educ. Adm. Q. 54, 501–528. doi: 10.1177/0013161X18769048

[B33] Liu S. Keeley J. W. Sui Y. Sang L. (2021). Impact of distributed leadership on teacher job satisfaction in China: the mediating roles of teacher autonomy and teacher collaboration. Stud. Educ. Eval. 71:101099. doi: 10.1016/j.stueduc.2021.101099

[B34] Liu Y. Werblow J. (2019). The operation of distributed leadership and the relationship with organizational commitment and job satisfaction of principals and teachers: a multi-level model and meta-analysis using the 2013 TALIS data. Int. J. Educ. Res. 96, 41–55. doi: 10.1016/j.ijer.2019.05.005

[B35] Lu X. (2024). Distributed leadership in Chinese higher education: conceptual understanding and barriers to its implementation. Educ. Manag. Adm. Leadersh. 52, 1352–1368. doi: 10.1177/17411432221145408

[B36] Meyer J. P. Allen N. J. (1991). A three-component conceptualization of organizational commitment. Hum. Resour. Manage. Rev. 1, 61–89. doi: 10.1016/1053-4822(91)90011-Z

[B37] Moè A. Katz I. (2022). Need satisfied teachers adopt a motivating style: The mediation of teacher enthusiasm. Learn. Individ. Differ. 99:102203. doi: 10.1016/j.lindif.2022.102203

[B38] Organisation for Economic Co-operation and Development (2025). TALIS 2024 Conceptual Framework. Paris: OECD Publishing.

[B39] Özdemir M. Küçükçene M. Abasli K. Pektaş V. Ayhan E. (2024). The effect of empowering leadership and teacher autonomy on affective commitment: the mediating role of teacher self-efficacy. Educ. Sci. 49, 201–224. doi: 10.15390/EB.2024.12663

[B40] Özdemir N. Kilinç A. Ç. Polatcan M. Turan S. Bellibaş M. S. (2023). Exploring teachers' instructional practice profiles: do distributed leadership and teacher collaboration make a difference? Educ. Admin. Quart. 59, 255–305. doi: 10.1177/0013161X231159092

[B41] Parker G. (2015). Teachers' autonomy. Res. Educ., 93, 19–33. doi: 10.7227/RIE.0008

[B42] Pearson L. C. Moomaw W. (2005). The relationship between teacher autonomy and stress, work satisfaction, empowerment, and professionalism. Educ. Res. Quart. 29, 38–54.

[B43] Podsakoff P. M. MacKenzie S. B. Podsakoff N. P. (2012). Sources of method bias in social science research and recommendations on how to control it. Annu. Rev. Psychol. 63, 539–569. doi: 10.1146/annurev-psych-120710-100452 21838546

[B44] Qian S. Zhang X. Liu J. (2024). Impacts of work-related rumination on employees' innovative performance: based on the conservation of resources theory. Balt. J. Manage. 19, 435–454. doi: 10.1108/BJM-01-2024-0023

[B45] Runhaar P. Konermann J. Sanders K. (2013). Teachers' organizational citizenship behavior: considering the roles of their work engagement, autonomy, and leader–member exchange. Teach. Teach. Educ. 30, 99–108. doi: 10.1016/j.tate.2012.10.008

[B46] Sha X. Chang Y. (2025). Occupational health and performance among Chinese university teachers: A COR theory model of health-promoting leadership and burnout. Eur. J. Investig. Health Psychol. Educ. 15:134. doi: 10.3390/ejihpe15070134 40709967 PMC12293935

[B47] Skaalvik E. M. Skaalvik S. (2014). Teacher self-efficacy and perceived autonomy: relations with teacher engagement, job satisfaction, and emotional exhaustion. Psychol. Rep. 114, 68–77. doi: 10.2466/14.02.PR0.114k14w0 24765710

[B48] Song Y. Hsieh C. C. (2026). Distributed leadership, teacher autonomy, and well-being: a multilevel analysis of novice teachers' teaching practice quality in Taiwan using TALIS 2018 data. Educ. Manag. Admin. Leadersh. doi: 10.1177/17411432251410038. [Epub ahead of print].

[B49] Spillane J. P. (2004). Educational leadership. Educ. Eval. Policy Anal., 26, 169–172. doi: 10.3102/01623737026002169

[B50] Spillane J. P. (2006). “Towards a theory of leadership practice: a distributed perspective,” in Rethinking Schooling, eds. I. Westbury, and G. Milburn (London: Routledge), 208–242.

[B51] Taxer J. L. Frenzel A. C. (2022). “The crucial role of teachers' emotions,” in Handbook of Social Influences in School Contexts, eds. K. R. Wentzel, and G. B. Ramani (London: Routledge), 243–258.

[B52] Walker A. Hu R. Qian H. (2012). Principal leadership in China: an initial review. Sch. Eff. Sch. Improv. 23, 369–394. doi: 10.1080/09243453.2012.678863

[B53] Wang G. Bai H. (2025). How does distributed leadership promote teachers' teaching innovation in China? a chain-mediated model of teaching autonomy and teaching efficacy. Asia-Pac. Educ. Res. 34, 2057–2069. doi: 10.1007/s40299-025-01014-9

[B54] Wang G. Bai H. Wang S. Hu Y. (2025). Teacher autonomy and teacher job satisfaction: a chain-mediated model of self-efficacy and intrinsic motivation. J. Psychol. Afr. 35:117. doi: 10.32604/jpa.2025.065785

[B55] Wang G. Bian J. Zhang H. (2026). Exploring the relationship between teaching autonomy and Chinese teachers' organizational commitment: a chain mediation model of self-efficacy and career well-being. Front. Psychol. 17:1820922. doi: 10.3389/fpsyg.2026.1820922 42137093 PMC13168076

[B56] Wright T. A. Hobfoll S. E. (2004). Commitment, psychological well-being, and job performance: an examination of conservation of resources (COR) theory and job burnout. J. Bus. Manag. 9, 389–406. doi: 10.1504/JBM.2004.141118

[B57] Yan X. Q. Zhou Y. Y. Zhang K. Cui G. Y. (2023). Perceived teacher enthusiasm and professional commitment: the mediating role of boredom and learning engagement. Psychol. Res. Behav. Manag. 16, 1149–1163. doi: 10.2147/PRBM.S400137 37069921 PMC10105573

[B58] Yao H. Ma L. (2024). Improving teacher career satisfaction through distributed leadership in China: the parallel mediation of teacher empowerment and organizational commitment. Int. J. Educ. Dev. 104:102960. doi: 10.1016/j.ijedudev.2023.102960

[B59] Zhang E. Ye Y. (2024). Understanding how to ignite teacher enthusiasm: the role of school climate, teacher efficacy, and teacher leadership. Curr. Psychol. 43, 13241–13254. doi: 10.1007/s12144-023-05387-2

